# AdipoRon, a new therapeutic prospect for Duchenne muscular dystrophy

**DOI:** 10.1002/jcsm.12531

**Published:** 2020-01-21

**Authors:** Michel Abou‐Samra, Camille M. Selvais, Raphael Boursereau, Sophie Lecompte, Laurence Noel, Sonia M. Brichard

**Affiliations:** ^1^ Endocrinology, Diabetes and Nutrition Unit, Institute of Experimental and Clinical Research, Medical Sector Université Catholique de Louvain Brussels Belgium

**Keywords:** Duchenne muscular dystrophy, AdipoRon, AMPK, Utrophin, Skeletal muscle, Inflammation

## Abstract

**Background:**

Adiponectin (ApN) is a hormone known to exhibit insulin‐sensitizing, fat‐burning, and anti‐inflammatory properties in several tissues, including the skeletal muscle. Duchenne muscular dystrophy (DMD) is a devastating disease characterized by dystrophin deficiency with subsequent chronic inflammation, myofiber necrosis, and impaired regeneration. Previously, we showed that transgenic up‐regulation of ApN could significantly attenuate the dystrophic phenotype in mdx mice (model of DMD). Recently, an orally active ApN receptor agonist, AdipoRon, has been identified. This synthetic small molecule has the advantage of being more easily produced and administrable than ApN. The aim of this study was to investigate the potential effects of AdipoRon on the dystrophic muscle.

**Methods:**

Four‐week‐old mdx mice (*n* = 6–9 per group) were orally treated with AdipoRon (mdx‐AR) for 8 weeks and compared with untreated (mdx) mice and to control (wild‐type) mice. *In vivo* functional tests were carried out to measure the global force and endurance of mice. *Ex vivo* biochemical and molecular analyses were performed to evaluate the pathophysiology of the skeletal muscle. Finally, *in vitro* tests were conducted on primary cultures of healthy and DMD human myotubes.

**Results:**

AdipoRon treatment mitigated oxidative stress (−30% to 45% for 4‐hydroxy‐2‐nonenal and peroxiredoxin 3, *P* < 0.0001) as well as inflammation in muscles of mdx mice (−35% to 65% for interleukin 1 beta, tumour necrosis factor alpha, and cluster of differentiation 68, a macrophage maker, *P* < 0.0001) while increasing the anti‐inflammatory cytokine, interleukin 10 (~5‐fold, *P* < 0.0001). AdipoRon also improved the myogenic programme as assessed by a ~2‐fold rise in markers of muscle proliferation and differentiation (*P* < 0.01 or less vs. untreated mdx). Plasma lactate dehydrogenase and creatine kinase were reduced by 30–40% in mdx‐AR mice, reflecting less sarcolemmal damage (*P* < 0.0001). When compared with untreated mdx mice, mdx‐AR mice exhibited enhanced physical performance with an increase in both muscle force and endurance and a striking restoration of the running capacity during eccentric exercise. AdipoRon mainly acted through ApN receptor 1 by increasing AMP‐activated protein kinase signalling, which led to repression of nuclear factor‐kappa B, up‐regulation of utrophin (a dystrophin analogue), and a switch towards an oxidative and more resistant fibre phenotype. The effects of AdipoRon were then recapitulated in human DMD myotubes.

**Conclusions:**

These results demonstrate that AdipoRon exerts several beneficial effects on the dystrophic muscle. This molecule could offer promising therapeutic prospect for managing DMD or other muscle and inflammatory disorders.

## Introduction

Duchenne muscular dystrophy (DMD) is the most frequently inherited human myopathy and the most devastating type of muscular dystrophy.^1^ DMD is caused by a defective gene that encodes for dystrophin, a key scaffolding protein that provides structural stability and integrity to muscle fibre membrane.^2^ Dystrophin‐deficient fibres are highly susceptible to injury, resulting in endless cycles of muscle necrosis and repair that lead to fibrosis and weakness.^1^, ^2^ Although dystrophin mutations represent the primary cause of DMD, it is the secondary processes involving persistent inflammation and subsequent impaired regeneration that likely exacerbate disease progression.^3^

Adiponectin (ApN) is a hormone abundantly secreted by adipocytes. It promotes insulin‐sensitizing, fat‐burning, and anti‐inflammatory/oxidative actions, thereby effectively counteracting several metabolic disorders, including type 2 diabetes, obesity, and cardiovascular disease.^4^, ^5^ ApN exerts its pleiotropic effects by modulating different signalling pathways in a variety of cell types,^5^ including the skeletal muscle.^6^

We have previously shown that ApN possesses powerful anti‐inflammatory effects on skeletal muscle exposed to acute (lipopolysaccharide injection)^7^ and chronic (obesogenic diet) inflammation.^8^ We have then tested the implication of ApN in DMD and found that mdx mice (a murine model of DMD) display low circulating ApN levels.^9^ When we crossed mdx mice with transgenic mice overexpressing ApN, we showed that ApN can act as preventive agent and delay disease progression. ApN up‐regulation was thus capable of reducing muscle inflammation/injury and improving force/myogenesis.^9^ Conversely, when we crossed mdx mice with ApN‐knockout mice, the resulting mdx mice with ApN depletion displayed lower global muscle force/endurance as well as increased muscle damage when compared with regular mdx mice. Muscular electrotransfer of the ApN gene counteracted all these abnormalities, indicating that ApN may be a powerful protector of the skeletal muscle.^10^ This protection was observed in all types of dystrophic skeletal muscle.^9^ It was mainly mediated through adiponectin receptor 1 (AdipoR1) and AMP‐activated protein kinase (AMPK) pathway, which led to suppression of nuclear factor‐kappa B (NF‐κB) activity and up‐regulation of utrophin (a dystrophin analogue).^9^, ^10^

However, the direct use of ApN as a therapeutic agent has several challenges. ApN possesses complex three‐dimensional structures, with its high molecular weight form being mainly required for its metabolic properties.^4^ In addition, ApN circulates at high concentrations in plasma, it turns over quickly, and, like every other peptide, it must be injected to produce its effects.^11^ Developing a new treatment mimicking the beneficial effects of ApN and that can be easily produced and administered is thus of great interest.^11^ AdipoRon, an orally active synthetic small‐molecule AdipoR agonist, has recently been proposed for the treatment of type 2 diabetes and other obesity‐related disorders in mice when usually given for a short time.^12^, ^13^, ^14^, ^15^, ^16^ Its long‐term effects have been scarcely studied,^12^ its potential anti‐inflammatory properties have not been tested in inflamed skeletal muscle, and its promyogenic properties have not yet been addressed.

The aim of this work was to explore whether AdipoRon may play a beneficial role in DMD. To this end, AdipoRon was orally administered for a period of 2 months, very early in mdx mice because muscle degenerative‐regenerative cycles begin as soon as 3–4 weeks of age.^17^, ^18^ We first examined whether treated mdx mice exhibited lower degree of skeletal muscle inflammation, oxidative stress, and injury together with improved muscular function, when compared with non‐treated mdx mice. We then unravelled the mechanisms underlying the beneficial effects exerted by AdipoRon. Finally, we tested some of the effects of this molecule *in vitro* on primary cultures of human myotubes derived from healthy and DMD subjects.

## Methods

### Animals

C57BL/10ScSn‐*Dmd* ^*mdx*^J mdx mice (murine model of DMD) and C57BL/10ScSnJ mice [used as wild‐type (WT) controls] were purchased from Jackson Laboratory (Maine, USA).

Three groups of male mice (*n* = 6–9 per group) were compared in our experiments. At 4 weeks old, each group of mice received gelatine, replacing the water bottles. The first group was WT mice, the second group consisted of untreated mdx mice (mdx), while the third group consisted of treated mdx mice (mdx‐AR). For this last group, a dose of 50 mg/kg/day of AdipoRon (AdipoGen, Kessel, Belgium) was added to the gelatine. Gelatines were replaced every other day over a period of 8 weeks.

Animals were maintained under a standard laboratory chow and housed at a constant temperature (22°C) with a fixed 12 h light to 12 h dark cycle (lights on from 7 a.m. to 7 p.m.). At the end of the treatment, 12‐week‐old mice were sacrificed either in the basal state or 1 week after *in vivo* functional tests. All mice were sacrificed between 09.00 and 11.00 h. Blood samples were saved. Pairs of *tibialis anterior* (TA) muscles were weighed, frozen in liquid nitrogen, and stored at −80°C for subsequent analyses.

### *In vivo* studies of global force or resistance

At 11 weeks old, mice were submitted to three main tests.

#### Wire test

Animals were suspended by their limbs from a 1.5‐mm‐thick, 60‐cm‐long metallic wire at 45 cm above soft ground. The time (seconds) until the mouse completely released its grasp and fell was recorded. Three trials were performed per session, with a 15 min recovery period between trials. The maximum time per trial was set to 180 s. For each mouse, the scores of the three trials were averaged.^19^

#### Grip test

The grip strength test measures the muscle strength of forelimb or of combined forelimb and hindlimb muscles. Limb strength was recorded using a grid connected to a sensor (Panlab‐Bioseb, Vitrolles, France). The mice were gently laid on the top of the grid so that their front paws (forelimb test) or both fore and hind paws (combined test) can grip the grid. Then mice were pulled back steadily until the grip was released down the complete length of the grid. Each test was repeated three times at an interval of 15 min. Results are presented as the mean of the three values of force recorded, related to body weight.^20^

#### Eccentric exercise

Mice were subjected to a downhill running exercise on a treadmill with a downward inclination of 15° and at a speed of 10 m/min for 10 min. This training was repeated daily for 3 days. Results represent the distance (metres) covered by each mouse on the third day, with a maximum distance of 100 m.^9^

### Quantification of muscle damage markers in plasma

Plasma creatine kinase (CK) and lactate dehydrogenase (LDH) activities were quantified to evaluate skeletal muscle damage as injured muscles release CK and LDH into the bloodstream at high levels. Kits were based on colorimetric methods and were used following the manufacturer's instructions (Gentaur, Kampenhout, Belgium). CK and LDH activities were expressed as IU/L.

### Light microscopy, immunohistochemistry, and morphometry

Tibialis anterior muscle samples were fixed in 10% formalin for 24 h and then embedded in paraffin. For immunohistochemistry, 5 μm sections were processed as previously described^10^ using rabbit polyclonal antibodies directed against peroxiredoxin 3 (PRDX3 dilution 1:500, incubation 2 h 30 min; gift from Bernard Knoops, University of Louvain, Brussels, Belgium^21^), 4‐hydroxy‐2‐nonenal (HNE, 1:100, 2 h 30 min), tumour necrosis factor alpha (TNFα, 1:100, 2 h 30 min), interleukin 1 beta (IL‐1β, 1:200, 2 h 30 min), and dystrophin (DYS, 1:100, 2 h 30 min) (all from Abcam, Cambridge, UK). Rat monoclonal antibodies directed against markers of macrophages [cluster of differentiation 68 (CD68), 1:100, 2 h 30 min; Abcam] and myosin heavy chain 7 (Myh7, 1:25, 2 h 30 min; Sigma‐Aldrich, Overijse, Belgium) were also used. Before immunostaining, sections were submitted to heat‐mediated antigen retrieval using a microwave oven and Tris‐citrate buffer (pH 6.5). Binding of antibodies was detected by applying for 30 min at room temperature a secondary antibody, which was a biotinylated goat anti‐rabbit IgG (H + L) or a biotinylated rabbit anti‐rat IgG (H + L) (Labconsult, Brussels, Belgium). Peroxidase activity was revealed with 3,3′‐diaminobenzidine (DAB) (Thermo Fisher Scientific, Aalst, Belgium), which produces a brown staining. For each marker, all slides were treated simultaneously for immunohistochemistry analysis and DAB revelation and then analysed together. Immunohistochemical controls were performed by omission of the first antibody or of the first and second antibodies or by using pre‐immune serum. Whole muscle sections were scanned using the Leica SCN400 slide scanner (Leica microsystems, Diegem, Belgium), and then the percentage of DAB surface area within muscle fibres was quantified using the Tissue Image Analysis 2.0 (Leica). The percentage of muscle fibres stained with Myh7 was determined on an average of 500 fibres per muscle section. In addition, 5‐μm‐thick sections were stained with haematoxylin (Labconsult) and eosin (Sigma‐Aldrich). The percentage of muscle fibres with centrally located nuclei, a characteristic feature of DMD, was calculated on more than 500 fibres per muscle section. Fibres cross‐sectional area was calculated on 200 fibres from four different fields per muscle section. Centrally located nuclei and cross‐sectional area were determined using the ImageJ 1.48v analysis software.

### Culture of human myotubes

Primary cultures of human skeletal muscle cells were initiated from myoblasts of DMD patients (*n* = 5; age range: 12–15 years) and healthy subjects (*n* = 3; age range: 15–17 years), which were provided by the French Telethon Myobank‐AFM (Association Française contre les Myopathies).

Cultures were performed as described.^22^ Myoblasts were grown in six‐well plates, at 5 × 10^4^ cells per well, at 37°C in the presence of 5% CO_2_ in F‐12 (Ham) supplemented with 20% foetal bovine serum, 1% l‐glutamine, and 1% penicillin‐streptomycin (all from Thermo Fisher Scientific). After 5–6 days of proliferation, at the end of which the seeding density had reached 70–80%, the growth medium was replaced by the fusion medium that consists of one part DMEM, one part F‐12 (Ham), 2% horse serum, 1% l‐glutamine, and 1% penicillin‐streptomycin (all from Thermo Fisher Scientific). This fusion medium was then changed every 2 days, and differentiation was allowed to continue for 12 days (time required to obtain mature myotubes with characteristic elongated and multinucleated morphology) before the experimentation period. Cells were always used at passages between 4 and 10. We have usually generated at least two independent cultures (i.e. run at different times and for each time, from a new vial of cryopreserved myoblasts) from a given biological donor. The donors were always chosen at random to avoid any bias of selection.

Twelve days after differentiation, cells were treated or not with human recombinant TNFα (10 ng/mL) + human interferon gamma (IFNγ) (10 ng/mL) and/or AdipoRon (25 μM) for 24 h (TNFα and IFNγ from PeproTech, Hamburg, Germany). In some experiments, cells were first transfected with either the On‐Targetplus Non‐targeting pool siRNAs (negative control, NT siRNAs, 50 nM) or a specific On‐Targetplus siRNA SMARTpool against human AdipoR1 (50 nM) using 10 μL Lipofectamine RNAiMAX reagent (all from Thermo Fisher Scientific) for 24 h. Next, the medium was renewed, and cells were treated with TNFα + IFNγ with AdipoRon for an additional 24 h. Knockdown efficiency was checked using real‐time quantitative PCR (RT‐qPCR), and mRNA expression level of AdipoR1 was decreased by up to 95%. At the end of the experiments, cells were collected and rinsed twice in cold PBS before RNA or protein extraction. For a given culture, experiments were always performed in duplicate, and the data obtained were then averaged.

### RNA extraction and real‐time quantitative PCR

RNA was isolated from mouse TA muscles and from cultured cells with TriPure reagent (Sigma‐Aldrich); 1 μg of total RNA was reverse transcribed, and 40 ng of total RNA equivalents were amplified using an iCycler iQ real‐time PCR detection system (Bio‐Rad Laboratories, Nazareth, Belgium). RT‐qPCR primers for mouse cyclophilin, interleukin 10 (IL‐10), myogenin (MyoG), myogenic regulatory factor 4 (Mrf4), myosin heavy chain 1 (Myh1) and 7 (Myh7) were used as reported.^10^ New mouse primer sequences were myogenic factor 5 (Myf5) (sense, 5′‐GAG GTG CAC CAC CAC CAA CCC‐3′; antisense, 3′‐CAT TCA GGC ATG CCG TCA GAG CAG‐5′) and myoblast determination protein 1 (MyoD) (sense, 5′‐CGC CGC TGC CTT CTA CGC AC‐3′; antisense, 3′‐GGG CCG CTG TAA TCC ATC ATG CC‐5′). RT‐qPCR primers for human TATA box‐binding protein, TNFα, and utrophin were similar to those previously reported.^9^ New human primer sequences were IL‐1β (sense, 5′‐GAA TCT CCG ACC ACC ACT ACA‐3′; antisense, 3′‐TGC ACA TAA GCC TCG TTA TCC C‐5′). The threshold cycles (Ct) were measured in separate tubes and in duplicate. The identity and purity of the amplified product were checked by electrophoresis on agarose minigels, and analysis of the melting curve was carried out at the end of the amplification. To ensure the quality of the measurements, each plate included a negative control for each set of primers.

### Protein extraction and ELISAs

Tibialis anterior muscle samples and cultured cells were homogenized in a lysis buffer supplemented with 1% protease/phosphatase inhibitor cocktail (Cell Signaling Technology, BIOKE, Leiden, The Netherlands). Proteins were quantified using the Bradford method and were stored at −80°C; 5–30 μg of total protein extracts were used for each analysis. ELISA assays were used to specifically detect and quantify HNE, TNFα, IL‐1β, IL‐10, the active phosphorylated form of AMPKα, the active phosphorylated form of the p65 subunit of NF‐κB, and utrophin A (UTRN) in the three groups of mice and in all cells cultured conditions (ELISAs for HNE, TNFα, IL‐1β, and IL‐10 from Abcam; ELISAs for P‐AMPK and for P‐p65 from Cell Signaling Technology; and ELISA for UTRN from Antibodies Online, Atlanta, USA). Kits were based on colorimetric methods and were carried out following manufacturer's instructions.

### Statistical analysis

Results are means ± standard deviation for the indicated number of mice or human subjects. When the three groups of mice (WT, mdx, and mdx‐AR) were compared, the effects of AdipoRon were assessed by one‐way analysis of variance followed by Tukey's test (Prism 8; GraphPad Software, California, USA). Comparisons between two myotubes conditions from a given subject were carried out using two‐tailed paired Student's *t*‐test (Prism 8). When four myotubes conditions were compared, the effects of AdipoRon and of inflammation were assessed by two‐way analysis of variance (raw paired data for each subject) followed by *post hoc* Sidak's multiple comparisons (Prism 8). Differences were considered statistically significant at *P* < 0.05.

### Study approval

All procedures conducted on mice were approved by the Ethical Committee for Animal Experimentation from the Medical Sector at Université Catholique de Louvain (no. LA1230396).

Primary cultures of human myotubes from DMD or healthy subjects were obtained via Myobank‐AFM (Association Française contre les Myopathies) in accordance with approved guidelines and regulations of the bioethics department of the Direction Générale de la Recherche et de l'Innovation, France (DGRI no. AC‐2013‐1868).

## Results

### Effects of AdipoRon treatment on muscle mass in mdx mice

AdipoRon was administered orally to mdx mice for a period of 2 months, starting from 4 weeks of age. Mdx littermates were thus separated into two groups: those who received a daily dose of AdipoRon at 50 mg/kg (mdx‐AR) and those who were left untreated (mdx). Both groups were also compared with untreated WT control mice. AdipoRon treatment did not affect the body weight of mdx mice (mdx: 30.3 ± 1.2 g vs. mdx‐AR: 29.5 ± 0.9 g; *n* = 9 per group, not significant). Because we have previously shown that ApN can alter the skeletal muscle mass,^9^ we examined whether AdipoRon could have a similar effect. As expected,^9^ mdx mice presented an increase in TA mass (mdx: 140 ± 8 mg vs. WT: 82 ± 5 mg; *n* = 9 per group, *P* < 0.0001), which was partially corrected in mdx‐AR mice (119 ± 5 mg, *n* = 9, *P* < 0.0001 vs. mdx and WT).

### Effects of AdipoRon treatment on muscle oxidative stress and inflammation in mdx mice

**Figure 1:**
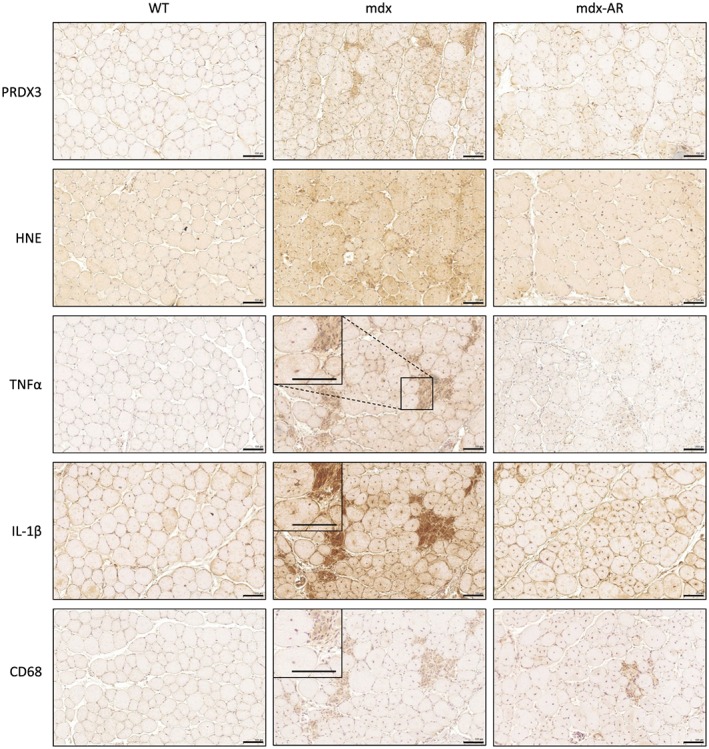
Effects of AdipoRon treatment on muscle oxidative stress and inflammation in mdx mice. Three groups of mice were compared at the age of 12 weeks: WT, mdx (untreated) and mdx‐AR (treated with AdipoRon) mice. Immunohistochemistry was performed on tibialis anterior (TA) with specific antibodies directed against two oxidative stress markers (PRDX3 and HNE), two pro‐inflammatory cytokines (TNFα and IL‐1β), and one macrophage marker (CD68). Representative sections for six mice per group are shown. Scale bar = 100 μm. Immunolabelling for inflammatory markers and for CD68 was performed on serial cross sections of TA muscles. Insets: Higher magnification of immunohistochemistry images (scale bar = 100 μm).

**Figure 2:**
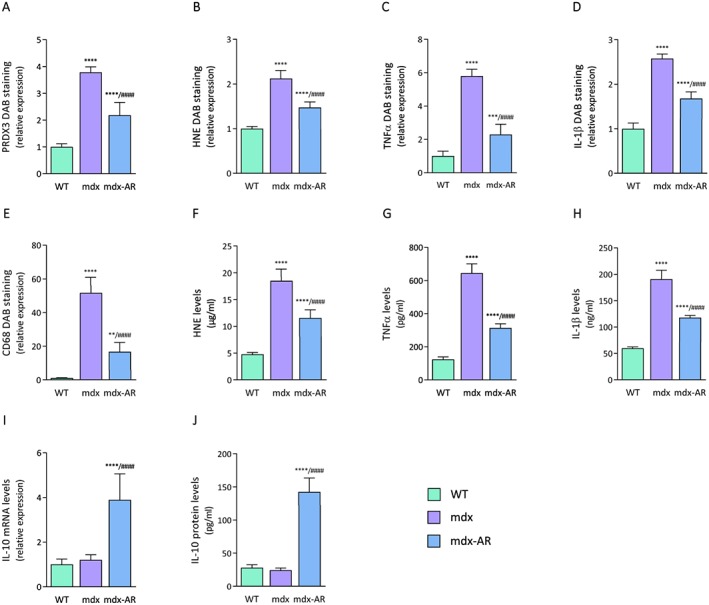
Quantification of markers of oxidative stress and inflammation in muscles of mdx mice after AdipoRon treatment. For each immunolabelling of *Figure* 1A–1E, the percentage of DAB deposit areas was calculated within muscle sections. The subsequent ratios were presented as relative expression compared with WT values. ELISA assays were used to quantify HNE (F), TNFα (G), and IL‐1β (H) in the three groups of mice. Results are represented as protein concentrations (pg/mL, ng/mL, or μg/mL). (I, J) mRNA and protein levels of IL‐10, an anti‐inflammatory cytokine. mRNA levels were normalized to cyclophilin, and the subsequent ratios were presented as relative expression compared with WT values. Protein levels were quantified by ELISA and expressed as pg/mL. Data are means ± standard deviation; *n* = 6 mice per group for all experiments. Statistical analysis was performed using one‐way analysis of variance followed by Tukey's test. ^**^ *P* < 0.01, ^***^ *P* < 0.001, ^****^ *P* < 0.0001 vs. WT mice. ^####^ *P* < 0.0001 vs. mdx mice.

First, we bluntly tested the hypothesis that oral administration of AdipoRon could slow down the progression of the dystrophic pathology by counteracting excessive inflammatory and oxidative reactions. When compared with WT mice, myofibers from TA muscles of mdx mice displayed a strong immunolabelling for PRDX3, a marker of oxidative stress, and for HNE, a lipid peroxidation product (*Figure* 1). Quantification of the immunolabelling showed that the extent of DAB staining within myofibers was ~4‐fold and 2‐fold higher in mdx than in WT mice, respectively (*Figure* 2A and 2B). Qualitatively similar results were observed for TNFα and for IL‐1β, two pro‐inflammatory cytokines (*Figure* 1), with an extent of DAB staining being ~6‐fold and 2.5‐fold greater in mdx than in WT mice, respectively (*Figure* 2C and 2D). In addition, a massive infiltration of macrophages (CD68, a marker of the pro‐inflammatory M1 phenotype^23^) was observed in mdx mice, but not in WT mice (*Figures* 1 and 2E). All these indicators of oxidative stress and inflammation were markedly reduced in mdx‐AR mice (*Figures* 1 and 2). These results were then validated by ELISA where all HNE, TNFα, and IL‐1β were found to be highly produced in skeletal muscles of mdx mice compared with WT mice, while their levels were decreased by 40–50% under AdipoRon treatment (*Figure* 2F–H). In addition, serial muscle cross sections revealed that both myocytes and macrophages contribute to the production of pro‐inflammatory cytokines. In mdx mice, areas that were positive for CD68 were also markedly positive for TNFα and IL‐1β, while both cytokines were produced by myofibers as well, yet to a lesser extent (*Figure* 1, insets). Remarkably, gene expression of IL‐10, an anti‐inflammatory cytokine that activates the M2 macrophage phenotype,^23^, ^24^ was also significantly increased in mdx‐AR mice, being four‐fold higher than in mdx mice (*Figure* 2I). Similarly, IL‐10 protein levels did not change between mdx and WT mice while it increased by over five‐fold in mdx‐AR mice (*Figure* 2J). Taken together, these data suggest that AdipoRon can strongly defend the skeletal muscle against excessive inflammatory responses and oxidative stress.

### Effects of AdipoRon treatment on muscle regeneration and composition in mdx mice

**Figure 3:**
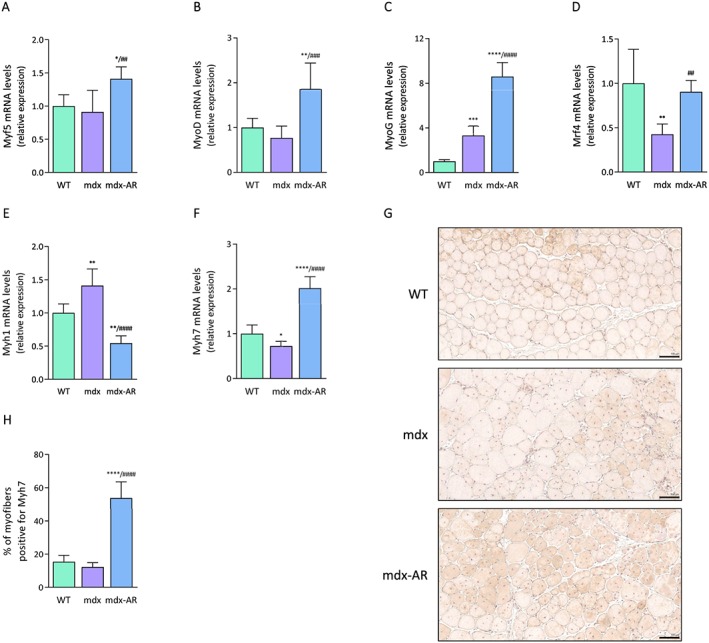
Effects of AdipoRon treatment on muscle regeneration and composition in mdx mice. mRNA levels of Myf5 (A) and MyoD (B), two markers of muscle proliferation. mRNA levels of MyoG (C) and Mrf4 (D), two markers of muscle differentiation. (E) mRNA levels of Myh1, a marker of fast‐twitch, glycolytic myofibers. (F) mRNA levels of Myh7, a marker of slow‐twitch, oxidative myofibers. mRNA levels were normalized to cyclophilin, and the subsequent ratios were presented as relative expression compared with WT values. (G) Immunolabelling for slow‐twitch myosin heavy chain (Myh7). Representative sections for six mice per group are shown. Scale bar = 100 μm. (H) Quantitation of myofibers expressing Myh7. Results are shown as % of type I myofibers. Data are means ± standard deviation; *n* = 6 mice per group for all experiments. Statistical analysis was performed using one‐way analysis of variance followed by Tukey's test. ^*^ *P* < 0.05, ^**^ *P* < 0.01, ^***^ *P* < 0.001, ^****^ *P* < 0.0001 vs. WT mice. ^##^ *P* < 0.01, ^###^ *P* < 0.001, ^####^ *P* < 0.0001 vs. mdx mice.

We have previously shown that ApN is capable of inducing myogenesis by activating key players in the skeletal myogenic programme.^9^, ^10^ Here we tested the effects of AdipoRon on the expression of two major factors of muscle proliferation, Myf5 and MyoD, and of two major factors of muscle differentiation, MyoG and Mrf4.^25^ There were no significant differences in the expression of Myf5 and MyoD between mdx and WT mice, while the expression of both factors was ~1.5‐fold to 2‐fold higher, respectively, in the TA muscles of mdx‐AR mice (*Figure* 3A and 3B). MyoG was increased (~3‐fold) in mdx compared with WT mice, but its expression was still further increased (~8.5‐fold) in mdx‐AR mice (*Figure* 3C). Conversely, the expression of Mrf4 was halved in mdx compared with WT mice but was restored to normal in mdx‐AR mice (*Figure* 3D). These data indicate a better muscle regeneration programme under AdipoRon treatment.

Furthermore, the expression of Myh1, a marker of fast‐twitch glycolytic type II fibres, was increased (~1.5‐fold) in mdx compared with WT mice but was markedly decreased in mdx‐AR mice (*Figure* 3E). A reverse pattern was observed for the expression of Myh7, a marker of slow‐twitch oxidative type I fibres (*Figure* 3F). Immunostaining for Myh7 revealed a wider labelling for mdx‐AR when compared with the other two groups (*Figure* 3G). The proportion of fibres with Myh7 staining was similar between WT and mdx mice (15% vs. 12%, respectively), while it significantly increased to 54% in mdx‐AR mice (*Figure* 3H). Together, these data indicate a switch towards a more resistant oxidative phenotype under AdipoRon treatment.

**Figure 4:**
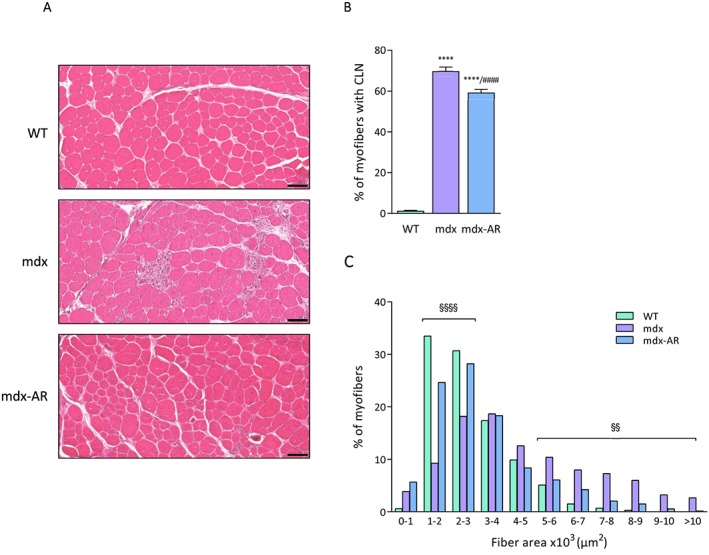
Effects of AdipoRon treatment on muscle morphometry in mdx mice. (A) Haematoxylin and eosin staining performed on TA sections from the three groups of mice. Scale bar = 100 μm. (B) Quantitation of myofibers with centrally located nuclei (CLN). Data are shown as % of myofibers with CLN. (C) Distribution of fibres cross‐sectional area (CSA). Data are expressed as % of myofibers. Data are means ± standard deviation; *n* = 6 mice per group for all experiments. Statistical analysis was performed using one‐way analysis of variance followed by Tukey's test. For (C), error bars were omitted for the sake of clarity. ^****^ *P* < 0.0001 vs. WT mice. ^####^ *P* < 0.0001 vs. mdx mice. ^§§^ *P* < 0.01, ^§§§§^ *P* < 0.0001 mdx mice vs. the two other groups.

Finally, the proportion of fibres with central nuclei, a hallmark of dystrophic muscle, was almost undetectable in WT mice (~1%), amounted to 70% in mdx mice and was reduced to 59% in mdx‐AR mice (*Figure* 4A and 4B). This reduction suggests an improved myogenic programme and a more complete muscle regeneration. In addition, mdx mice presented an increase in the percentage of large myofibers when compared with WT and mdx‐AR mice [≥5.10^3^ μm^2^, *P* < 0.01 vs. these two groups (*Figure* 4C)]. In contrast, mdx‐AR mice showed a larger number of small myofibers than untreated mdx (≤3.10^3^ μm^2^, *P* < 0.0001 vs. mdx mice) and exhibited a distribution profile shifted to the left, as in WT mice (*Figure* 4C). This result indicates that AdipoRon treatment increases and normalizes the number of small diameter fibres, which are known to have an oxidative phenotype.

### Effects of AdipoRon treatment on AMPK signalling in mdx mice

**Figure 5:**
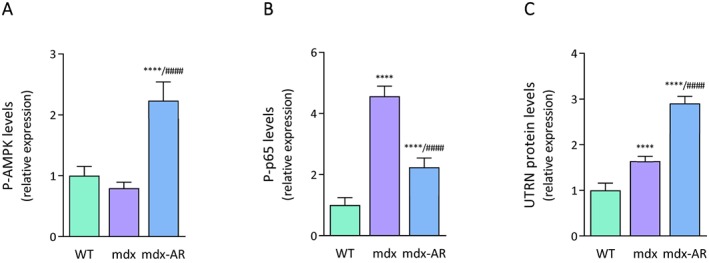
Effects of AdipoRon treatment on AMPK activity, NF‐κB activity, and utrophin A levels in mdx mice. ELISA assays were used to quantify the active phosphorylated form of AMPKα (P‐AMPK) (A), the active phosphorylated form of the p65 subunit of NF‐κB (P‐p65) (B), and utrophin A (UTRN) (C) in the three groups of mice. Absorbance data were presented as relative expression compared with WT values. Data are means ± standard deviation; *n* = 6 mice per group for all experiments. Statistical analysis was performed using one‐way analysis of variance followed by Tukey's test. ^****^ *P* < 0.0001 vs. WT mice. ^####^ *P* < 0.0001 vs. mdx mice.

Because AdipoRon activates AMPK by binding to AdipoR1 in muscle, thereby triggering beneficial effects on the metabolic syndrome,^12^, ^26^ and because AMPK is involved in skeletal muscle remodelling,^27^, ^28^ we explored the activation of this vital kinase. The phosphorylated and active form of AMPK (P‐AMPK) was found to be similar in WT and mdx mice, while being two‐fold higher in mdx‐AR mice (*Figure* 5A). Furthermore, because AMPK signalling was previously found to repress NF‐κB activity,^9^ and because NF‐κB regulates muscle inflammation as well as muscle regeneration/degeneration,^3^, ^29^, ^30^ we tested the effect of AdipoRon on this pleiotropic transcription factor. The phosphorylated and active form of the p65 subunit (P‐p65) was significantly higher (~4.5‐fold) in mdx compared with WT mice but was then halved in mdx‐AR mice (*Figure* 5B). Lastly, AMPK is also a powerful inducer of the expression of utrophin A (UTRN), a dystrophin analogue that can help rescue the dystrophic phenotype.^9^, ^31^ Accordingly, protein levels of UTRN, which were slightly augmented in mdx mice likely to compensate the lack of dystrophin, were further up‐regulated in mdx‐AR mice (~3‐fold vs. WT) (*Figure* 5C). It is worth noting that almost no revertant fibres expressing dystrophin were detected in either untreated or treated mdx mice (Supporting Information, *Figure* *S1*). Taken together, these data indicate a significant activation of the AMPK signalling pathway, in AdipoRon‐treated mdx mice, leading to down‐regulation of NF‐κB activity and up‐regulation of utrophin.

### Effects of AdipoRon treatment on muscle injury in mdx mice

**Figure 6:**
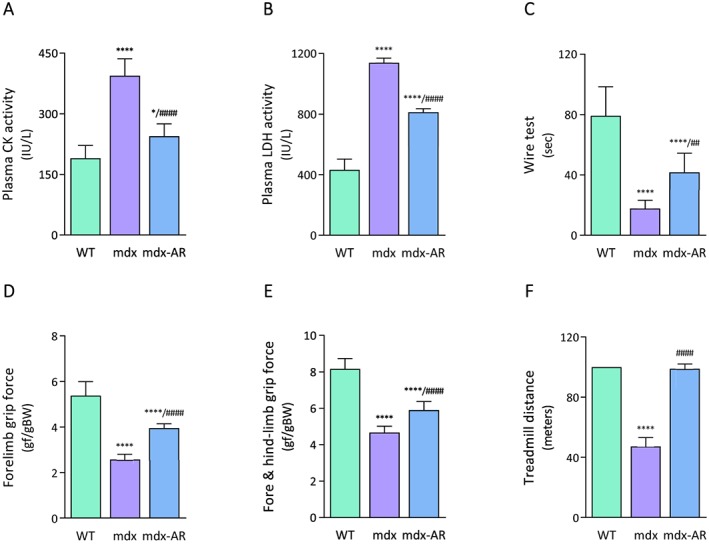
Effects of AdipoRon treatment on muscle injury and function in mdx mice. Muscle injury was assessed, in non‐exercised mice, by plasma activity of CK (A) and LDH (B) expressed as IU/L. Data are means ± standard deviation; *n* = 6 mice per group for both experiments. Functional in vivo studies were carried out in mice from the three groups. (C) Mice were subjected to a wire test where they were suspended by their limbs and the time until they completely released the wire and fell was recorded (seconds). Mice were lowered on a grid connected to a sensor to measure the muscle force of their forelimbs (D) or of both their forelimb and hindlimb (E); data were then expressed in gram force relative to body weight (gf/gBW). (F) Mice were submitted to a downhill treadmill exercise for 10 min during three consecutive days. On the third session, the covered distance (metres) was measured for each mouse, 100 m being the maximal distance. Data are means ± standard deviation; *n* = 9 mice per group for all in vivo tests. Statistical analysis was performed using one‐way analysis of variance followed by Tukey's test. ^*^ *P* < 0.05, ^****^ *P* < 0.0001 vs. WT mice. ^##^ *P* < 0.01, ^####^ *P* < 0.0001 vs. mdx mice.

Muscle damage was assessed by measuring the plasma activity of CK and LDH. Elevation of both CK and LDH in circulation indicates a breach in the muscle membrane and is thus associated with injury. CK and LDH activities, measured herein, were ~2.5‐fold and ~3‐fold higher in mdx than in WT mice, respectively, while both activities declined by over 40% and 30% in mdx‐AR mice, respectively (*Figure* 6A and 6B). Hence, AdipoRon treatment is likely to significantly decrease sarcolemmal injury in the skeletal muscle.

### Effects of AdipoRon treatment on global force and resistance of mdx mice

In order to evaluate the effects of AdipoRon on muscle function, mice were subjected to three *in vivo* functional tests: the wire test, the grip test, and a treadmill exercise. The wire test gives an indication on muscle force and resistance to fatigue. In this test, the time during which the mouse is suspended on a horizontal wire is measured.^19^ Mdx mice fell down much quicker than WT mice, while mdx‐AR mice showed intermediate resistance (*Figure* 6C). The grip test measures strength of forelimb or of combined forelimb and hindlimb muscles.^20^ The force developed by forelimbs of mdx mice was over 50% lower than that of WT mice, while it was partially rescued by AdipoRon treatment (*Figure* 6D). Similar results were observed for the combined forelimb and hindlimb force (*Figure* 6E). Finally, resistance to fatigue was further evaluated by an eccentric treadmill. On the last day (third) of exercise, WT mice covered the maximum distance (100 m),^9^ the running distance of mdx mice fell drastically (~45 m), while that of mdx‐AR mice was not statistically different from that of WT ones (*Figure* 6F). These data indicate enhanced dystrophic muscle force and endurance under AdipoRon treatment.

### Effects of AdipoRon treatment on inflammation‐challenged human myotubes

**Figure 7:**
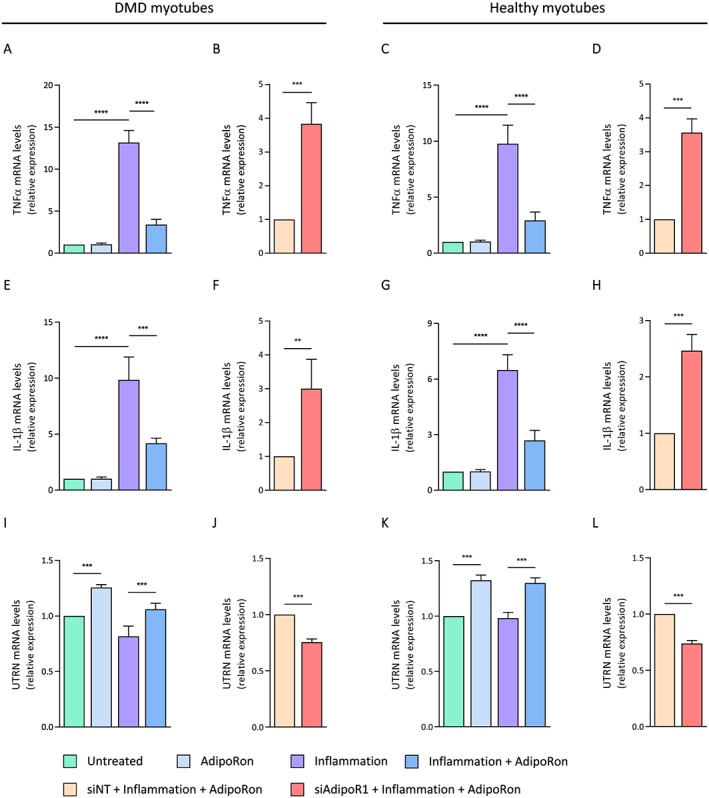
Effects of AdipoRon treatment on human myotubes challenged by pro‐inflammatory cytokines. (A–D) mRNA levels of TNFα, (E–H) mRNA levels of IL‐1β, and (I–L) mRNA levels of utrophin A in primary culture of human DMD or healthy myotubes. Cells were treated with or without AdipoRon (25 μM), while being challenged or not with TNFα (10 ng/mL) and IFNγ (10 ng/mL) for 24 h. In some conditions (B, D, F, H, J, L), cells were first transfected (24 h) with siRNA against AdipoR1 (50 nM) or a negative [non‐targeting, siNT (50 nM)] control. mRNA levels were normalized to human TATA box‐binding protein. The subsequent ratios are presented as relative expression compared with basal conditions (i.e. no inflammation and no AdipoRon, represented by green columns) or to siNT conditions (orange columns). For DMD myotubes, data are means ± standard deviation; *n* = 5 cultures from five different DMD patients. For healthy myotubes, data are means ± standard deviation; *n* = 6 independent cultures from three different healthy subjects (each run twice at two different time points and for each time, from a new vial of cryopreserved myoblasts). Each patient or subject is represented as its own control. Statistical analysis was performed on raw paired data, using two‐tailed Student's *t*‐test (B, D, F, H, J, L) and two‐way analysis of variance followed by Sidak's multiple comparisons test (A, C, E, G, I, K). ^**^ *P* < 0.01, ^***^ *P* < 0.001, ^****^ *P* < 0.0001 for indicated conditions.

Finally, we tested the direct anti‐inflammatory effects of AdipoRon in primary cultures of DMD and healthy human myotubes. In order to mimic the inflammatory micro‐environment of DMD, we challenged the myotubes for 24 h with an inflammatory stimulus (TNFα/IFNγ) and tested the expression of two major pro‐inflammatory cytokines, TNFα and IL‐1β, which are significantly up‐regulated in murine and human dystrophic muscles.^9^, ^32^, ^33^ These results are summarized in left and middle panels coloured in green to sustained blue (*Figure* 7A, 7C, 7E, and 7G). As expected, the expression of both cytokines was highly induced under inflammatory conditions (third vs. first column), while AdipoRon treatment significantly reduced their expression (fourth vs. third column) in both DMD (*Figure* 7A and 7E) and healthy myotubes (*Figure* 7C and 7G). In addition, AdipoRon was capable of significantly increasing utrophin A gene expression in both DMD and healthy myotubes in normal (second column) and inflammatory (fourth column) conditions (*Figure* 7I and 7K). These beneficial effects of AdipoRon on gene expression were abolished by siRNA silencing of the AdipoR1 gene (panels in orange and red: *Figure* 7B, 7D, 7F, 7H, 7J, and 7L).

**Figure 8:**
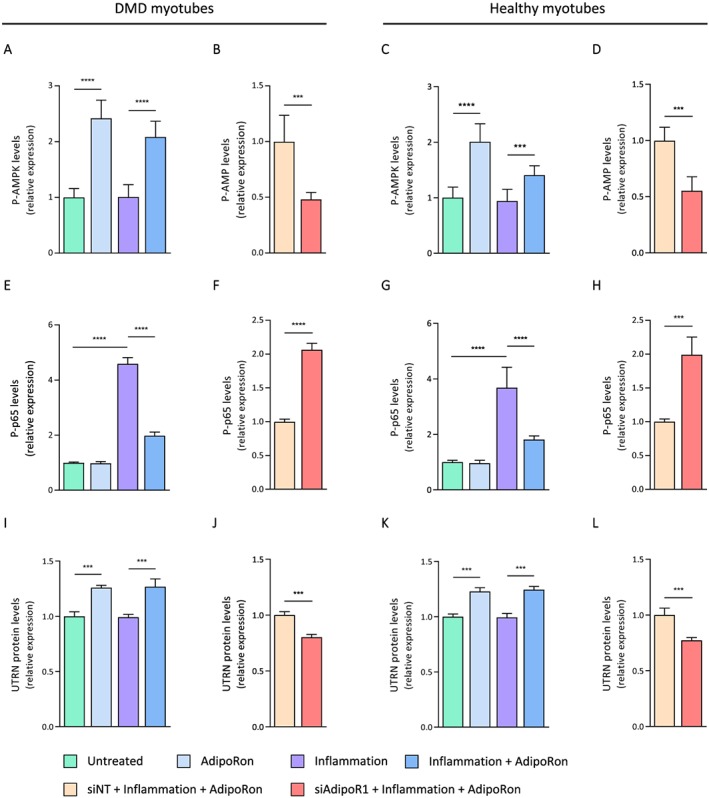
Effects of AdipoRon treatment on AMPK activity, NF‐κB activity, and utrophin A levels in human myotubes challenged by pro‐inflammatory cytokines. ELISA assays were used to quantify the active phosphorylated form of AMPKα (P‐AMPK) (A–D), the active phosphorylated form of the p65 subunit of NF‐κB (P‐p65) (E–H), and utrophin A (UTRN) (I–L) in primary culture of human DMD or healthy myotubes. Cells were treated with or without AdipoRon (25 μM), while being challenged or not with TNFα (10 ng/mL) and IFNγ (10 ng/mL) for 24 h. In some conditions (B, D, F, H, J, L), cells were first transfected (24 h) with siRNA against AdipoR1 (50 nM) or a negative [non‐targeting, siNT (50 nM)] control. Absorbance data are presented as relative expression compared with basal conditions (i.e. no inflammation and no AdipoRon, represented by green columns) or to siNT conditions (orange columns). For DMD myotubes, data are means ± standard deviation; *n* = 5 cultures from five different DMD patients. For healthy myotubes, data are means ± standard deviation; *n* = 6 independent cultures from three different healthy subjects (each run twice at two different time points and for each time, from a new vial of cryopreserved myoblasts). Each patient or subject is represented as its own control. Statistical analysis was performed on raw paired data, using two‐tailed Student's *t*‐test (B, D, F, H, J, L) and two‐way analysis of variance followed by Sidak's multiple comparisons test (A, C, E, G, I, K). ^***^ *P* < 0.001, ^****^ *P* < 0.0001 for indicated conditions.

Furthermore, we evaluated the activation of the AMPK signalling pathway in human myotubes. The phosphorylated and active form of AMPK (P‐AMPK) was found to be similar between basal and inflammatory conditions (first and third columns), while being 2‐fold to 2.5‐fold higher under AdipoRon treatment (second and fourth columns) in both DMD (*Figure* 8A) and healthy myotubes (*Figure* 8C). Moreover, the phosphorylated and active form of the p65 subunit (P‐p65) was significantly higher (4‐fold to 4.5‐fold) in inflammatory conditions compared with normal ones (third vs. first column) but was then halved under AdipoRon treatment (fourth vs. third column) (*Figure* 8E and 8G). Lastly, protein levels of UTRN were also up‐regulated (~30%) after only 24 h of AdipoRon treatment (*Figure* 8I and 8K). Again, all these beneficial effects of AdipoRon were abolished after silencing of the AdipoR1 gene (*Figure* 8B, 8D, 8F, 8H, 8J, and 8L). Hence, AdipoR1 appears to be necessary for the anti‐inflammatory of action of AdipoRon on human myotubes. This action is again mediated by AMPK signalling that leads to repression of NF‐κB as well as up‐regulation of utrophin A.

## Discussion

In recent years, we have been interested in the beneficial effects of ApN in DMD. We and others have shown that mdx mice display low circulating levels of ApN.^9^, ^34^ Replenishment of ApN by transgenesis mitigated the dystrophic phenotype by attenuating inflammation while improving regeneration, muscle function, and physical performance.^9^ Likewise, increasing serum ApN by moderate exercise was associated with improved muscle function in mdx mice.^35^ On the contrary, complete depletion of ApN levels greatly aggravated the phenotype.^10^ As yet, it is still unclear whether circulating ApN levels are also decreased in DMD patients. Two studies^34^, ^36^ have measured plasma ApN in Duchenne boys, but both were lacking an appropriate control group and did not—or only partially—take into account the several confounding factors that may influence these levels (puberty stage, fat mass and insulin resistance, current treatment, etc.).^4^ When considering DMD muscle, myotubes from these patients definitely produced less ApN than those from controls.^22^ Thus, there could be a rationale to therapeutically correcting the low levels of ApN in dystrophic patients.

Although bioactive recombinant ApN appears to have beneficial effects, its use faces several hurdles. Indeed, ApN has the disadvantage of being difficult to produce and administer.^11^ We thus wanted to test whether AdipoRon, an ApN receptor small‐molecule agonist,^12^ could have beneficial properties on the dystrophic muscle. Several teams have already highlighted the potential protective effects of this molecule in various pathologies related to type 2 diabetes, obesity, and other associated disorders such as liver dysfunction, diabetic nephropathy, cardiovascular disease, or depression.^12^, ^13^, ^14^, ^15^, ^16^, ^37^, ^38^ One novel interest of this study was to test the effects of AdipoRon on inflammation and on myogenesis in the skeletal muscle and to potentially offer a new and exciting therapeutic prospect for managing DMD.

By orally treating mdx mice for 8 weeks with AdipoRon (mdx‐AR), we showed that this AdipoR agonist could protect the skeletal muscle against excessive inflammatory reactions and oxidative stress. This treatment decreased the expression of pro‐inflammatory cytokines and the number of M1 macrophages. AdipoRon could therefore promote macrophage polarization towards an anti‐inflammatory M2 phenotype, as highlighted by the decreased expression of CD68 (a M1 marker) and the increased expression of IL‐10, which activates the switch to the tissue‐healing M2 phenotype.^24^ Oxidative stress markers were also decreased in mdx mice treated with AdipoRon. These results are in agreement with our previous work carried out on transgenic mdx mice with chronic overexpression of ApN, where ApN up‐regulation helped rescue the dystrophic phenotype.^9^ Likewise, ApN added to cultured myotubes of DMD patients induced a shift in the secretion of downstream myokines towards a less inflammatory profile.^22^ Herein, we show that oral administration of a small synthetic molecule, such as AdipoRon, can also be key in counterbalancing excessive inflammatory and oxidative responses in the dystrophic muscle.

AdipoRon also positively affected the muscle regeneration process. Indeed, the skeletal muscle of treated mdx mice displayed increased expression of positive factors of the proliferative stage as well as of the differentiation phase.^25^ Moreover, mdx‐AR mice displayed a significant decrease in the percentage of muscle fibres with central nucleated nuclei. Taken together, these data indicate a more efficient skeletal myogenic programme after AdipoRon treatment. This myogenic effect agrees with studies showing that adiponectin or its globular form (gApN) can positively affect several features of the muscle regeneration programme. Firstly, gApN could activate satellite cells (muscle stem cells) to exit quiescence and elicit a motility programme, driving them to reach the injury site.^39^ Secondly, gApN enhances C2C12 differentiation, through increased expression of several muscle markers, and provokes cell fusion and myofiber formation.^40^ Thirdly, blocking ApN *in vivo* suppresses stem cell proliferation in muscles from exercise‐trained mice.^41^

These beneficial effects are highlighted by the significant reduction in mdx‐AR mice of both plasma CK and LDH, reflecting less fibre membrane damage in the dystrophic muscle. Moreover, this could also explain the reduced skeletal muscle mass in treated mdx mice, due to less immune cells infiltration and to changes in muscle composition that favour the smaller, oxidative fibres. Accordingly, AdipoRon treatment greatly improved the *in vivo* global muscular force and exercise endurance of mdx mice. The present results are also consistent with the improvement of exercise endurance observed in diet‐induced obese mice treated with AdipoRon for 10 days.^12^

We then wanted to explore the main mechanisms of action of AdipoRon. In treated mdx mice, AMPK was found to be overactivated, as demonstrated by the increased phosphorylated form of AMPK. Activation of this pathway appears to be important for rescuing the dystrophic phenotype.^9^, ^42^ Firstly, AMPK can be a powerful suppressor of NF‐κB signalling and of inflammation,^43^ a pivotal process whose ablation improves the dystrophic muscle, as seen here after AdipoRon treatment as well as in numerous other studies.^3^, ^44^, ^45^, ^46^ Secondly, AMPK pathway leads to the activation of peroxisome proliferator‐activated receptor‐γ coactivator 1 alpha (PGC‐1α), a key regulator of energy metabolism.^47^ PGC‐1α, via mitochondrial biogenesis, can contribute to the change of muscle fibre type towards an oxidative phenotype, which is more resistant to the absence of dystrophin.^9^, ^27^, ^48^ Moreover, utrophin A, a dystrophin analogue, is a target gene of PGC‐1α,^9^, ^28^ and up‐regulation of utrophin may restore sarcolemmal integrity and confer morphological and functional improvements in mdx mice.^31^, ^49^ Our results demonstrate that AdipoRon can be strong activator of AMPK signalling, which can suppress NF‐κB activity, increase muscle oxidative capacity, and up‐regulate utrophin, thus helping to rescue the dystrophic phenotype. Compared with other pharmacological agents targeting the AMPK pathway in mice,^50^ the improvements afforded by AdipoRon are not only impressive (see the dramatic improvement in the *in vivo* running test) but also more extensive. Indeed, AdipoRon can exert a plethora of beneficial effects on several target tissues (heart, adipose tissue, kidney, liver, and brain) through both AMPK‐dependent and AMPK‐independent mechanisms.^12^, ^13^, ^14^, ^16^, ^38^

Because of its convenience and cost‐effectiveness, the mdx mouse remains the most widely used model for studying DMD.^51^ Although this mouse has a point mutation in the DMD gene and a non‐functional dystrophin, it exhibits a milder phenotype than patients.^52^ It was thus important to validate our data in human myotubes from DMD subjects. So far, the direct muscular effects of AdipoRon *in vitro* have only been studied on mouse C2C12, myotubes.^12^ We thus extended these data and demonstrated that AdipoRon recapitulated the effects observed in mice in primary cultures of either healthy or DMD myotubes. Similar results were previously obtained with ApN treatment.^9^, ^22^ Yet, in some tissues, ApN and AdipoRon could act through different pathways to elicit similar effects. Thus, for example, in smooth muscle, the action of AdipoRon is mediated by mechanisms that were totally different from those of ApN.^15^

To date, glucocorticoids (GCs) are the only medication commonly used to help delay the progression of DMD. GCs are believed to be effective in dystrophy because of their anti‐inflammatory and immunosuppressive properties.^53^, ^54^ However, their chronic use is hampered by several side effects.^55^, ^56^ Those include weight gain and growth retardation, vertebral fractures and muscle atrophy, behaviour disorder, and cushingoid facies in DMD boys. Other side effects involve glucose intolerance, hypertension, and gastrointestinal problems.^53^, ^55^, ^57^ AdipoRon could well be a strong alternative to GCs due to its powerful anti‐inflammatory action on the skeletal muscle. In addition, AdipoRon has the advantage of improving muscle regeneration, increasing utrophin expression, and favouring a resistant oxidative muscle phenotype. Furthermore, AdipoRon appears to be safe in mice: it did not impair but rather protected liver and renal function.^13^, ^38^ Moreover, unlike GCs, AdipoRon enhanced insulin sensitivity,^12^ prevented obesity,^16^ hypertension,^15^ and mood disturbances,^16^ and even increased the lifespan of obese mice.^12^

So far, a few clinical trials with oral compounds targeting some of the pathways triggered by AdipoRon are underway. However, the data currently available are limited because of the small number of patients enrolled (i.e. five patients for the AMPK activator, metformin^58^) or the short‐term duration of the treatment (i.e. 1 week for the NF‐κB inhibitor, CAT‐1004^59^). Trials with the utrophin up‐regulator (Ezutromid®) turned out to be disappointing and have been recently stopped (http://www.musculardystrophynews.com). To date, AdipoRon (or adiponectin) has not been tested in humans yet. However, we feel that this molecule exhibiting potent pleiotropic effects has a crucial role to play in attenuating the consequences of the lack of dystrophin.

Furthermore, AdipoRon could also benefit viral vector‐based DMD gene therapy such as exon‐skipping or micro‐dystrophin cDNA transfer.^60^, ^61^, ^62^ By mitigating the dystrophic micro‐environment (strengthening leaky membranes and reducing inflammation and oxidative stress), pre‐treatment with AdipoRon could avoid the subsequent loss of virus‐mediated genomes occurring in diseased muscle early after vector injection.^61^, ^63^ AdipoRon and DMD gene therapy could thus exert synergistic effects.

## Conclusions

Collectively, these data unarguably prove that AdipoRon, a small AdipoR agonist, can greatly attenuate the dystrophic phenotype in mdx mice. While dystrophin gene therapy constitutes the best hope for treating DMD,^62^ we propose that AdipoRon could provide additional benefits to complete and improve upon already existing treatments. More specifically, AdipoRon could be a valid option to GCs. Similarly, AdipoRon could also pave the way for novel strategies to treat other muscle and inflammatory disorders.

## Author contributions

S.M.B. conceived and designed the study. S.L. conducted pilot studies with AdipoRon and initiated this work. M.A.‐S., S.L., and L.N. performed mice crossing and AdipoRon treatment. M.A.‐S. and R.B. performed *in vivo* studies on mice as well as sample extraction. M.A.‐S. and C.M.S. performed immunohistochemistry, qPCR, ELISA, and assay kits on mouse tissues. M.A.‐S. and L.N. conducted the culture of human myotubes and RT‐qPCR analysis. M.A.‐S. performed ELISA tests on mouse tissues and on human myotubes. M.A.‐S. and S.M.B. analysed and interpreted the results. M.A.‐S. and S.M.B. wrote the manuscript. All authors have read, edited, and given approval of the final version of the manuscript.

## Conflict of interest

None declared.

## Materials and correspondence

Correspondence and material requested should be addressed to Michel Abou‐Samra (michel.abousamra@uclouvain.be).

## Supporting information

Figure S1 Effects of AdipoRon treatment on revertant fibers in mdx mice. Three groups of mice were compared at the age of 12 weeks: WT, mdx (untreated) and mdx‐AR (treated with AdipoRon) mice. Immunohistochemistry was performed on Tibialis anterior (TA) with specific antibodies directed against dystrophin (DYS). Representative sections for 6 mice per group are shown. Scale bar = 100 μm.
