# Supplementary material for: AdipoRon, a new therapeutic prospect for Duchenne muscular dystrophy
Source: J Cachexia Sarcopenia Muscle. 2020 Jan 21;11(2):518–33. doi: 10.1002/jcsm.12531 (PMC7113498; doi:10.1002/jcsm.12531)
Supplement: Supplementary file 1 — Figure S1 Effects of AdipoRon treatment on revertant fibers in mdx mice. Three groups of mice were compared at the age of 12 weeks: WT, mdx (untreated) and mdx‐AR (treated with AdipoRon) mice. Immunohistochemistry was performed on Tibialis anterior (TA) with specific antibodies directed against dystrophin (DYS). Representative sections for 6 mice per group are shown. Scale bar = 100 μm. [file JCSM-11-518-s001.pdf]

Fig.S1.

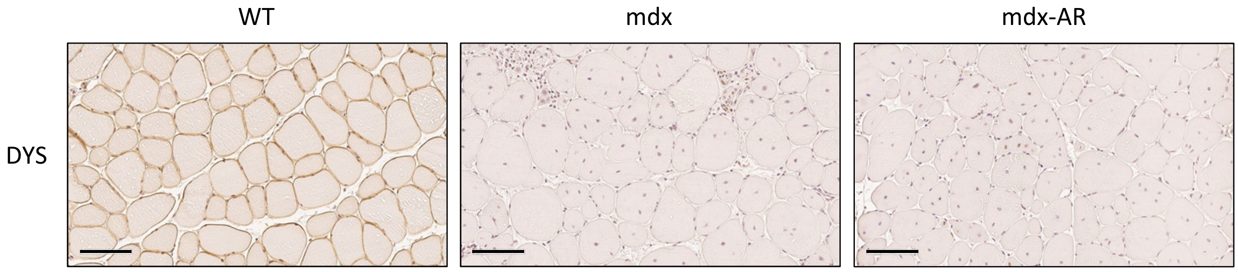

Fig.S1. Effects of AdipoRon treatment on revertant fibers in mdx mice. Three groups of mice were compared at the age of 12 weeks: WT, mdx (untreated) and mdx-AR (treated with AdipoRon) mice. Immunohistochemistry was performed on *Tibialis anterior* (TA) with specific antibodies directed against dystrophin (DYS). Representative sections for 6 mice per group are shown. Scale bar = 100 µm.
